# Diagnosis and management of hyponatraemia: AGREEing the guidelines

**DOI:** 10.1186/s12916-015-0277-8

**Published:** 2015-02-13

**Authors:** Alexander P Maxwell

**Affiliations:** School of Medicine, Dentistry and Biomedical Science, Queen’s University Belfast, 11 South Office, Regional Nephrology Unit, Belfast City Hospital, Belfast, BT9 7AB UK

**Keywords:** Hyponatraemia, Guidelines, Systematic review

## Abstract

Hyponatraemia is a common electrolyte disorder associated with significant complications and controversies regarding its optimal management. Clinical practice guidelines and consensus statements have attempted to provide clinicians with evidence-based diagnostic and treatment strategies for hyponatraemia. Recently published guidance documents differ in their methods employed to review the quality of available evidence. Nagler et al. used the Appraisal of Guideline for Research and Evaluation (AGREE II) instrument in a systematic review of guidelines and consensus statements for the diagnosis and management of hyponatraemia. Nagler and colleagues highlighted the variability in methodological rigour applied to guideline development and inconsistencies between publications in relation to management of hyponatraemia (including the recommended rate of correction of a low serum sodium concentration). These differences could cause confusion for practising physicians managing patients with hyponatraemia.

Please see related article: http://www.biomedcentral.com/1741-7015/12/231.

## Background

Hypotonic hyponatraemia (serum sodium concentration <135 mmol/L with low osmolality) is the most common electrolyte abnormality in hospitalised adult patients [1]. The diagnosis and management of hyponatraemia may be complex, costly, and controversial. Hyponatraemia has diverse aetiologies and is additionally defined clinically by its duration, “acute” (<48 hours) versus “chronic” (>48 hours), and by the presence or absence of symptoms. Management can be challenging particularly in the emergency setting where different treatment options may help or indeed harm individual patients. It is perhaps surprising that management of this electrolyte disorder has a limited evidence base in part due to the paucity of high quality randomised controlled trials. Multiple clinical practice and consensus guidelines for the diagnosis and management of hyponatraemia have been published by local, national, and international organisations. These guidelines represent genuine efforts to address the diagnostic challenges and controversies in its management, particularly in relation to the rate of correction for the serum sodium concentration. The systematic review by Nagler et al. [2], reviewing the quality of recent published guidelines and consensus statements for diagnosis and treatment of hyponatraemia, has highlighted important variations in both their development and recommendations.

### Guidelines of hyponatraemia treatment

Hypotonic hyponatraemia is a clinical state where there is a relative excess of water to sodium content in the extracellular fluid [3]. Acute hyponatraemia is clinically important as it can cause significant morbidity and mortality associated with rapid development of symptomatic cerebral oedema. Prompt treatment to raise the serum sodium concentration in this setting is life-saving. Chronic hyponatraemia, even if asymptomatic, is associated with many adverse outcomes including prolonged hospitalisation, gait instability, falls, fractures, and increased bone loss [4,5]. Diverse strategies to correct chronic hyponatraemia have been recommended and success of such treatment is dependent on the underlying aetiology for hyponatraemia. Overly rapid correction of chronic hyponatraemia may trigger an osmotic demyelination syndrome resulting in serious neurological deficits and death [6].

The methodological quality of guideline development and consensus statements can be assessed using the Appraisal of Guidelines for Research and Evaluation (AGREE II) process [7]. This tool is used to systematically evaluate six guideline domains including scope, stakeholder involvement, editorial independence, rigour of development, clarity, and applicability. Nagler et al. identified five clinical practice guidelines and five consensus statements after a comprehensive search of English and non-English publications, guideline databases, and professional society websites [2]. Their recommendations differed with respect to classification of hyponatraemia, diagnostic tests, doses of saline to use for correction, limits for the rise in serum sodium concentration, and the most appropriate second line therapies for management. The overall quality of these publications (measured by the AGREE tool) was mixed.

Should we be surprised that individual hyponatraemia guidelines “failed” this test of quality? Arguably, the diagnosis and management of hyponatraemia cannot be subjected to this sort of rigorous analysis because of the low level of evidence available to help various expert panels and guideline groups write internationally consistent advice. For example, hyponatraemia occurring within 48 hours is an arbitrary cut-off for determining the presence of acute hyponatraemia, the desired rate of correction for hyponatraemia in most settings is not universally agreed, availability of certain recommended therapies is country dependent and impacted by differing regulatory indications for drugs, and even the biochemical threshold for defining hyponatraemia varies widely in the literature. Furthermore, many of the clinical algorithms require initial assessment of the extracellular fluid volume to determine if a hyponatraemic patient is hypovolaemic, euvolaemic, or hypervolaemic. In practice, this physical examination of fluid balance can be subject to misinterpretation if the clinical signs are subtle.

The applicability of guidelines remains a major issue. The target audience are the clinicians who may infrequently encounter an ill patient with either acute symptomatic hyponatraemia requiring urgent correction or a patient with profound chronic hyponatraemia and additional risk factors (malnourished, hypokalaemia, history of alcoholism) for the osmotic demyelination syndrome. It is improbable that clinicians would always be able to quickly access or indeed assimilate the comprehensive advice in scholarly recent publications on diagnosis and treatment of hyponatraemia. For instance, Spasovski et al. (European Guideline Development Group) produced a 39 page clinical practice guideline [8] and Verbalis et al. (Expert Panel Recommendations) published an extensive narrative review running to 42 pages [9]. These guidelines are freely available but have several important differences, particularly in relation to the drug treatment of chronic hyponatraemia, which may lead to some confusion for clinicians. If appropriate, fluid restriction is used in the management of chronic hyponatraemia but this is frequently of limited efficacy. Additional pharmacological agents have been used, including demeclocycline, lithium, urea, loop diuretics, and vaptan drugs (conivaptan and tolvaptan) [8,9]. Tolvaptan has been used more extensively in the USA for the treatment of hypervolaemic and euvolaemic hyponatraemia compared to Europe, where tolvaptan’s licence is restricted to hyponatraemia caused by the syndrome of inappropriate anti-diuretic hormone [10]. The European clinical practice guideline has been widely endorsed by European specialist societies for nephrologists, endocrinologists, and intensive care medicine clinicians.

In the “real world”, the non-expert doctor who initially recognises and responds to severe hyponatraemia (serum sodium concentration <120 mmol/L) in a critically ill patient will often be a junior trainee working “out of routine office hours”. In this emergency setting, the doctor may have limited immediate access to important additional diagnostic tests, e.g., serum and urine osmolality and urine electrolytes. Urgent treatment decisions may need to be taken to manage symptoms such as confusion and seizures, with incomplete patient history and diagnostic information. There is a general consensus that hypertonic saline is effective in the immediate management of acute symptomatic hyponatraemia but available guidelines differ on the volumes and rates of saline infusion. Ultimately, it is clinical judgement rather than adherence to a particular guideline that will determine an individual patient’s treatment. Intuitive clinical algorithms, with proven efficacy, would help to encourage “best practice” in the diagnosis and management of hyponatraemia.

## Conclusions

Improving both the accuracy of diagnosis and the appropriate management of hyponatraemia are important goals given the morbidity and mortality associated with this common electrolyte disorder. A greater consistency in future clinical practice guidelines would represent a significant educational achievement and, crucially, would help clinicians to pick the best options for patients with hyponatraemia.
